# Newborn Screening for Li-Fraumeni Syndrome: Patient Perspectives

**DOI:** 10.21203/rs.3.rs-4351728/v1

**Published:** 2024-05-14

**Authors:** Makenna Beigh, Jennie Vagher, Rachel Codden, Luke D. Maese, Sabina Cook, Amanda Gammon

**Affiliations:** University of Utah School of Medicine; Huntsman Cancer Institute at the University of Utah; Division of Epidemiology, Department of Internal Medicine, University of Utah; Primary Children’s Hospital; Utah Department of Health and Human Services; Huntsman Cancer Institute at the University of Utah

**Keywords:** Li-Fraumeni syndrome (LFS), Newborn screening, Cancer predisposition syndrome, Pediatric cancer, Psychosocial implications, Ethical considerations

## Abstract

**Background:**

Li-Fraumeni syndrome (LFS) is an inherited cancer predisposition syndrome with an estimated prevalence of 1 in 3,000–5,000 individuals. LFS poses a significant cancer risk throughout the lifespan, with notable cancer susceptibility in childhood. Despite being predominantly inherited, up to 20% of cases arise *de novo*. Surveillance protocols facilitate the reduction of mortality and morbidity through early cancer detection. While newborn screening (NBS) has proven effective in identifying newborns with rare genetic conditions, even those occurring as rarely as 1 in 185,000, its potential for detecting inherited cancer predispositions remains largely unexplored.

**Methods:**

This survey-based study investigates perspectives toward NBS for LFS among individuals with and parents of children with LFS receiving care at single comprehensive cancer center in the U.S.

**Results:**

All participants unanimously supported NBS for LFS (n = 24). Reasons included empowerment (83.3%), control (66.7%), and peace of mind (54.2%), albeit with concerns about anxiety (62.5%) and devastation (50%) related to receiving positive results. Participants endorsed NBS as beneficial for cancer detection and prevention (91.7%), research efforts (87.5%), and family planning (79.2%) but voiced apprehensions about the financial cost of cancer surveillance (62.5%), emotional burdens (62.5%), and insurance coverage and discrimination (54.2%). Approximately 83% of respondents believed that parental consent should be required to screen newborns for LFS.

**Conclusion:**

This study revealed strong support for NBS for LFS despite the recognition of various perceived benefits and risks. These findings underscore the complex interplay between clinical, psychosocial, and ethical factors in considering NBS for LFS from the perspective of the LFS community.

## Introduction & Background

Li-Fraumeni syndrome (LFS) is an autosomal dominant cancer predisposition syndrome that arises from germline pathogenic variants (PVs) in the tumor suppressor gene *TP53* [1]. The prevalence of LFS is estimated to be between 1 in 3,000 to 1 in 5,000 [2], with some studies estimating a prevalence as high as 1 in 500 [3] in specific regions. LFS is regarded as one of the most penetrant hereditary predisposition syndromes. Affected individuals face lifetime cancer risks nearing 70% for men and 95% for women with a 50% chance of developing multiple primary malignancies. [4]. It poses considerable cancer risks during childhood, with an approximate 20% chance of developing cancer by age 20, which is significantly higher than that of the general population at 0.3% [4–6]. A wide range of cancer types have been noted in the context of LFS with most cancers comprising brain tumors, pre-menopausal breast cancer, sarcomas of the soft tissue and bone, and adrenocortical carcinoma (ACC) [7]. While LFS is commonly inherited from an affected parent, up to 20% of LFS cases are *de novo* [5]. These individuals often lack a significant family history and are diagnosed following one or more cancer diagnoses.

To mitigate the impact of these cancers, recommended surveillance protocols for individuals of any age with LFS aim to identify and treat malignancies at early stages, thereby minimizing both mortality and morbidity. A variety of institutions around the world have put forth surveillance recommendations for individuals with LFS with the goal of identifying malignancies at early stages [8–11]. In general, surveillance methods involve frequent imaging studies, laboratory tests, and clinical examinations to monitor a variety of at-risk organs. Given the substantial risk for early-onset cancers and multiple primary cancers, coupled with the existence of early detection surveillance, there is a compelling need to identify individuals with LFS before cancer onset.

Newborn screening has demonstrated success in identifying newborns with genetic conditions before the onset of symptoms, allowing early intervention and treatment [12]. Screening relies predominantly on biochemical testing to identify at-risk infants but often utilizes genetic testing during diagnostic evaluation following a positive screen. The United States Department of Health and Human Services (DHHS) puts forth a national Recommended Uniform Screening Panel (RUSP) for specific disorders based on evidence of the potential net benefit of screening, the ability to screen for the disorder and the availability and accessibility of effective treatments. Initiating treatment early during the neonatal period and ideally before symptom onset can prevent mortality and long-term damage, although certain conditions may manifest at a later stage in childhood rather than during infancy. Each state is encouraged to screen for all disorders on RUSP but may implement its own screening panel with fewer or more conditions included [13, 14].

While screening newborns for pediatric cancer predisposition syndromes is not currently included on RUSP, preliminary model-based studies highlight potential survival and economic benefits associated with newborn screening for pediatric cancer predisposition syndromes in the U.S., including LFS [15–17]. Real-world efforts to screen newborns for LFS have been studied in Parana, Brazil, where a prevalent *TP53* PV (R337H) is found in 1 in 350 individuals [18]. This PV is associated with distinctive cancer risks, notably a heightened susceptibility to early childhood ACC and pre-menopausal breast cancer [19]. Screening for this variant in all newborns of Parana, Brazil resulted in the initiation of surveillance for ACC among the screen-positive infants. Through surveillance efforts, the children who developed ACC were diagnosed in early stages and cured surgically with minimal morbidity [18–20]. Many of these infants lacked an LFS-like family history, indicating that family history alone may not effectively identify infants at risk [18]. Together, these studies suggest that LFS could be a promising candidate for newborn screening in the U.S.

While evidence of the clinical and economic benefits of newborn screening for LFS emerges, social and ethical considerations must also be considered. The psychosocial implications of receiving an LFS diagnosis through newborn screening, particularly in the absence of a notable family history, present a complex terrain. The unique psychosocial landscape of LFS has been well documented, owing to high lifetime cancer risks and the frequency and intensity of cancer surveillance efforts [21–25]. However, overall perceptions of newborn screening for LFS are not well documented, especially amongst those within the LFS community. This study aims to (1) explore the perspectives of individuals diagnosed with or parents of children diagnosed with LFS regarding newborn screening for LFS and (2) identify the factors that are most important when considering newborn screening for LFS.

## Methods

### 1. Study Design & Instrument

This survey-based study employed a cross-sectional design to explore participant perspectives of newborn screening for LFS. The survey instrument was developed through review of the literature and input from genetic counselors working in oncology and newborn screening. The instrument was reviewed and revised to ensure adherence to best practices in questionnaire development [26]. The survey was built and disseminated with Research Electronic Data Capture (REDCap) software. The initial draft was then piloted by an additional genetic counselor to assess clarity, relevance, and comprehensibility.

The survey comprised three total domains: demographic information, personal and family history of LFS and cancer, and overall perspectives related to screening newborns for LFS. A brief paragraph about the purpose and current procedures of newborn screening was provided as well as additional information about LFS, including *de novo* rates and general surveillance recommendations. Participants were asked to imagine that they were considering screening their newborn for LFS and were encouraged to reflect on personal experiences as well as hypothetical experiences of a parent who may be in this position. Participants were encouraged to provide open-ended responses to provide greater detail about their personal and family histories, diagnostic experiences, or any other topics covered in the survey. The survey instrument is provided in **Supplementary Material 1**.

### 2. Participants

Participants were recruited from a single institution, Huntsman Cancer Institute at the University of Utah, and were identified from a prior research initiative in which individuals provided consent to be approached for subsequent research efforts.

### 3. Eligibility and Exclusion Criteria

Eligibility criteria included enrollment in the previous study, having a personal molecular diagnosis of LFS or being a parent of a child with a molecular diagnosis of LFS, being at least 18 years of age, and being proficient in English. Those who were under 18 years of age, non-English-speaking, deceased, lacking a PV or likely PV in *TP53*, lacking an available email address, not enrolled in our institution’s previous research study or for whom enrollment status was unclear were excluded.

### 4. Recruitment

Participants were recruited from an institutional database, ensuring a comprehensive representation of the LFS community seen at our institution. Recruitment strategies included electronic distribution of the survey via email.

### 5. Procedure

Participants were invited to complete the survey via email, with a clear explanation of the study’s purpose and a link to the online survey platform. Completion of the survey would qualify participants to be entered in a drawing to receive monetary compensation. Informed consent was implied electronically by the completion of the survey. Data collection occurred between January 9th, 2024 and February 9th, 2024. Reminders were sent to non-respondents at three distinct time points to enhance participation.

### 6. Ethical Approval

This study received ethical approval from the University of Utah Institutional Review Board (00165563). Confidentiality of participant data was maintained by storing data on a password-protected computer and storing participant identifiers separately from the coded survey data.

### 7. Data Analysis

Descriptive statistics were used to summarize survey data. Qualitative data from open-ended survey responses were coded by the primary investigator using an open-coding approach and reviewed by an additional investigator to ensure concordance and data saturation.

## Results

### 1. Participant Characteristics

**Figure 1:**
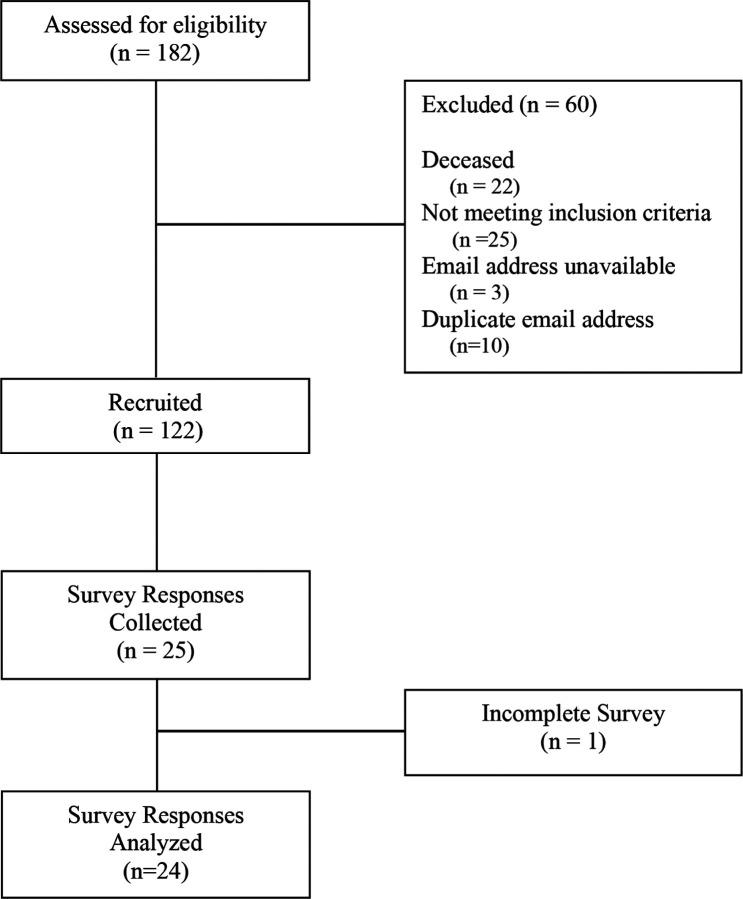
Recruitment Flowchart

A total of 122 individuals with unique email addresses were identified as meeting inclusion criteria. Of those, 24 participants completed the survey, resulting in a 19.67% response rate (Fig. 1). Most individuals were between 26 and 65 years of age, female-identifying, white/non-Hispanic, and insured through their or another peron’s employer. Detailed demographic characteristics are presented in Table 1.

### 2. Personal History Information

Among all respondents, 91.7% (N=22) had a personal diagnosis of LFS. Fourteen respondents (63.7%) reported a personal history of cancer. A total of 35 distinct primary cancers were reported, with breast cancer being the most common cancer type (N=12, 34.3%). Approximately 9% (N=3) of respondents with cancer were diagnosed with their first cancer in childhood. Additional personal history information is presented in Table 2.

### 3. Family History Information

Two-thirds (66.7%) of all respondents reported having biological children. Most (N=14, 87.5%) respondents with children reported that all their children had undergone molecular testing for LFS. The majority of these individuals (N=12, 75%) reported having at least one child diagnosed with LFS, with 70% (N=14) reporting that their children were diagnosed by age 12. One individual reported in an open-ended response that they utilized *in vitro* fertilization with preimplantation genetic diagnosis to conceive children unaffected by LFS. All children with LFS reportedly inherited it from a parent.

Among respondents who had biological children diagnosed with LFS, one-quarter (N=3) reported having children with at least one cancer diagnosis. There were six distinct instances of cancer reported, most of which were diagnosed before the age of 12 years. One respondent did not enter the age of their child’s first cancer diagnosis but reported a common childhood malignancy. Other classic LFS-related childhood cancers were reported. All respondents whose children had both LFS and cancer reported that their child was diagnosed with cancer before being diagnosed with LFS. Table 3 presents data related to participants’ children. LFS and cancer history information related to additional first- and second-degree relatives is presented in **Supplementary Material 2.**

### 4. Perspectives of Newborn Screening for LFS

All respondents indicated support for newborn screening for LFS (N=24). Reasons included the belief that knowledge is power (N=20, 83.3%), the potential to reduce uncertainty (N=16, 66.7%), the sense of control it provides (N=16, 66.7%), and the peace of mind it offers (N=13, 54.2%). Respondents indicated that in the setting of a newborn screen result that diagnosed their child with LFS they would feel both anxious (N =15, 62.5%), devastated (N=12, 50%) and/or guilty (N=10, 41.7%). Conversely, all respondents (N=24) indicated they would feel relieved if their child screened negative, and 62.5% (N=15) would feel happy.

The majority (N=18, 75.0%) of respondents viewed a hypothetical positive newborn screen as an opportunity to prepare for their child’s future, including initiation of the most appropriate medical surveillance plan and cancer prevention efforts (N= 22, 91.7%). Respondents also viewed newborn screening as an opportunity for future family planning (N=19, 79.2%) and research efforts (N=21, 87.5%). Overall, 87.5% (N=21) of respondents agreed that screening was in the best interest of their child. However, the majority (58.3%, N=14) expressed concerns about potential anxiety for the child. The most commonly identified concerns regarding newborn screening for LFS were related to the financial cost of cancer surveillance (N=15, 62.5%) and insurance coverage and discrimination (N=13, 54.2%), with one participant citing concerns that “…most insurances will not cover the proper screenings, now I have run into certain hospitals not even accepting my insurance, thus not being able to afford screening and skipping needed scans.” The emotional burden of regular cancer surveillance was also a frequently cited concern (N=15, 62.5%). Lastly, a significant proportion (83.3%, N=20) of respondents believed parental consent should be required to screen newborns for LFS. Detailed information regarding participant perspectives on newborn screening for LFS is provided in Table 4.

## Discussion

This study represents an original effort to gather insights from the LFS community regarding newborn screening for LFS. Participants uniformly expressed strong support for newborn screening for LFS despite recognizing various perceived risks. Research utilizing simulated U.S. birth cohort data has consistently highlighted both clinical and economic advantages of identifying newborns with pediatric-onset cancer predisposition syndromes. For instance, Yeh et al. demonstrated that screening newborns for eleven genes associated with pediatric cancer predisposition syndromes, including *TP53*, could lead to a remarkable 53.5% reduction in cancer-related deaths through adherence to surveillance protocols, with a 7.8% reduction in cancer deaths in the first 20 years of life [15]. Similarly, another study aimed at assessing the clinical and economic merits of universal newborn screening for LFS in a simulated population specifically reported a noteworthy 7.2% decrease in cancer-related deaths within the first 20 years of life. It’s worth noting that these benefits may be underestimated given that the study focused solely on four childhood-onset cancers and omitted evaluation of adult-onset cancers, some of which may manifest before the age of 20, such as breast cancer [16]. Both studies concluded that efforts to screen newborns for pediatric cancer syndromes are cost-effective, especially as sequencing costs continue to decrease. Additionally, the clinical and economic advantages of newborn screening for cancer predisposition syndromes may also be applied to other at-risk relatives through cascade screening [17].

As the use of clinical genetic testing for inherited cancer predisposition continues to expand and our understanding of the true prevalence of LFS and its associated features evolves, clearer insights into the utility of newborn screening for LFS may emerge. For example, the Brazilian R337H founder PV is associated with a unique clinical profile that differs from that of classic LFS with clear age-specific cancer risks but lower overall lifetime risks [19]. R337H represents just one of over a thousand *TP53* pathogenic and likely PVs associated with LFS. Recent research efforts have sought to elucidate the connection between the clinical range of LFS and various variant-level molecular mechanisms at play [27]. As the spectrum of genotype-phenotype correlations continues to emerge, a better understanding of specific cancer risks and ages of onset may inform newborn screening practices.

The psychosocial implications of receiving an LFS diagnosis through newborn screening, particularly in the absence of a notable family history, are important to consider. The results of this study reiterate that coping with the emotional, psychological, and social aspects of an LFS diagnosis at birth are important factors. In our sample of individuals with LFS, parental anxiety, devastation, and guilt were commonly identified as anticipated responses to a positive screen. In general, the psychosocial experience of receiving positive newborn screen results regardless of the condition involves a range of parental and familial emotions, including shock, guilt, anxiety, and denial among others [28,29]. Respondents also indicated that the result would likely cause anxiety for the affected child, highlighting psychological implications for the individual screened. A systematic review assessing the psychological impact of genetic information on children found little conclusive evidence indicating damaging psychological outcomes among those who received genetic risk information, although the conditions investigated were not exclusively associated with pediatric cancer [30].

Though not addressed in this study, the autosomal dominant nature of LFS likely carries greater psychosocial implications for additional family members compared to the many autosomal recessive conditions commonly screened for in newborns. Few participants anticipated difficulty sharing their child’s screening results with extended family, highlighting the potentially complex nature of communication within families about health information. While participants in this study identified screening as an opportunity for informed family planning, it may also raise challenges for future reproductive decisions, further contributing to the psychosocial impact [31]. Additionally, while there is ample evidence that the opportunity for proactive cancer surveillance provides individuals with a sense of control and relief following negative screening, as reiterated in this study, cancer-related anxiety around the time of imaging, colloquially known as “scanxiety” [32], has also been a reported barrier to adhering to surveillance protocols and positive health behaviors [33]. This phenomenon is aptly reflected in respondents’ concerns about the emotional burden related to their child undergoing periodic cancer surveillance. The extent of such a burden may be magnified by having multiple at-risk children and having a personal LFS diagnosis. Despite the anticipated emotional toll, participants valued the utility of newborn screening for LFS. Overall, the psychosocial experiences outlined above may not be unique to LFS, but the distinctive clinical profile of LFS may exacerbate the psychosocial components of newborn screening for this condition.

More broadly, the topic of high throughput sequencing (HTS) as a first-tier test for newborn screening appears to be both controversial and of growing interest in the medical and public health communities. Numerous ethical, legal, social, and psychological concerns have been raised in addition to the many technical, economic, and medical challenges identified [34]. The adoption of expansive HTS-based testing in newborn screening mirrors similar methodologies employed in diagnostic evaluations for critically ill newborns, infants, and children of varying ages. The possibility of receiving unexpected findings on newborn screening resembles the experience of encountering incidental or secondary findings in genomic diagnostic testing. Currently, the American College of Genetics and Genomics (ACMG) suggests that pathogenic and likely PVs in 81 genes, including *TP53*, associated with actionable medical conditions be disclosed as secondary findings, provided consent is obtained from the parents or guardian of a minor undergoing such testing [35]. Studies have shown that parents are often interested in receiving these results citing motivations related to early intervention or surveillance, the ability to prepare for the future, and implications for the family [36–38], many of the important factors revealed in the current study about newborn screening.

Because parental consent is necessary for the disclosure of secondary findings for whole-exome or whole-genome sequencing, it is a vital factor to consider when implementing NGS methods for newborn screening. Some states, including the state in which the current study was performed, require that the site of collection assumes responsibility for both collecting initial specimens and educating expectant parents about newborn screening’s purpose, procedures, opt-out options, and obtaining consent [39]. Typically, consent is verbal, with no requirement for written parental consent. Debates exist over whether HTS-based newborn screening necessitates formal informed consent with some arguing it is unnecessary if the screened conditions offer clear medical benefits in childhood [40–42]. This study found that most participants support the requirement for explicit parental consent in newborn screening for LFS, underscoring the need to consider diverse viewpoints on formal consent processes.

Insurance and discrimination implications persist for any information gleaned from genetic testing. This study revealed widespread concern among participants regarding genetic discrimination. While the Genetic Information Non-Discrimination Act (GINA) prohibits health insurers and employers from such discrimination, life, disability, and long-term care insurance falls outside its coverage [43]. Screening initiatives targeting variable-onset conditions like LFS may affect the availability and cost of these types of insurance for newborns, as policies are not typically designed for this demographic. Moreover, while health insurance is protected by GINA, individual insurance, and institutional policies may heighten the financial strain of adhering to the frequent and often expensive surveillance protocols recommended for individuals with LFS, especially if surveillance is initiated at birth. Though not assessed in this study, it is anticipated that financial and psychosocial concerns intensify when surveillance uncovers concerning findings that necessitate additional evaluation, a scenario frequently encountered by those at elevated risk of cancer.

## Conclusion

In conclusion, this study sheds light on the perspectives of the LFS community regarding newborn screening for LFS and reveals strong support for its perceived benefits despite the recognized risks. Our findings provide additional insight beyond the clinical and economic advantages of identifying newborns with cancer predisposition syndromes. Real-world screening efforts in regions like Parana, Brazil, provide compelling evidence of the feasibility and effectiveness of such programs. However, it is imperative to prioritize comprehensive approaches that balance clinical benefits with ethical, social, and psychological considerations to ensure the well-being of affected individuals and families. Continued research and dialogue in this field will be essential for advancing evidence-based practices and policies that optimize the potential of newborn screening for LFS and similar conditions while mitigating potential risks and disparities.

## Limitations & Future Research

Several limitations are pertinent to this study. First, we recognize that individuals with known LFS would not be the population to benefit most from an initiative to screen newborns in the general population. However, we chose to specifically sample the LFS community rather than the general population given their insights derived from personal and familial experiences with LFS, cancer, and cancer surveillance. Second, the available population for this research was limited to a single institution, potentially impacting the diversity and representativeness of the sample. Third, the response rate to the survey was low, which may be a reflection of self-selection bias, affecting the reliability of the findings. Lastly, the survey instrument used in this study was not validated. These limitations should be considered when interpreting the results and implications of the study.

Moving forward, future research endeavors should strive to expand beyond the confines of our sampled population, which consisted of individuals already impacted by LFS. While their perspectives are invaluable, they may not comprehensively capture the impact of implementing newborn screening for LFS on a general population scale. Exploring the viewpoints of unaffected individuals without known family history would offer broader insights into the societal implications surrounding screening for pediatric cancer predisposition syndromes like LFS. Moreover, understanding the perspectives of healthcare providers, including genetic counselors, oncologists, and clinicians involved in newborn screening, is crucial for assessing feasibility, ethical concerns, and potential challenges in implementation and follow-up. Additionally, investigating the utility of newborn screening for other pediatric-onset conditions, such as inherited arrhythmias or cardiomyopathies, presents an intriguing avenue for future research. Lastly, supplementing quantitative data with qualitative research could enrich our understanding by delving deeper into the nuanced perspectives, experiences, and concerns of various stakeholders involved in newborn screening initiatives.

## Figures and Tables

**Table 1.** Participant Demographics

	n (%)
**Age**	
18–25 years	2 (8.3%)
26–40 years	10 (41.7%)
41–65 years	11 (45.8%)
66+ years	1 (4.2%)
**Sex assigned at birth**	
Female	18 (75.0%)
Male	6 (25.0%)
Intersex	0 (0.0%)
Other	0 (0.0%)
**Current gender identity**	
Female	17 (70.8%)
Male	6 (25.0%)
Non-binary	1 (4.2%)
Agender	0 (0.0%)
Transgender	0 (0.0%)
Other gender	0 (0.0%)
**Race** (select all that apply)	
White/non-Hispanic	23 (95.8%)
White/Hispanic	1 (4.2%)
Asian/Pacific Islander	0 (0.0%)
Black	0 (0.0%)
American Indian	0 (0.0%)
Alaska Native	0 (0.0%)
Other	1 (4.2%)
South Asian	1 (100.0%)
**Current insurance status** (select all that apply)	
Insured through my employer or another person’s employer	21 (87.5%)
Insured through health insurance marketplace	1 (4.2%)
Insured through a military-related agency	1 (4.2%)
Insured through Medicare/Medicaid	2 (8.3%)
Not currently insured	0 (0.0%)
Other	0 (0.0%)
**Highest level of education**	
Less than high school degree	0 (0.0%)
High school degree	1 (4.2%)
Some college/vocational degree	7 (29.2%)
College degree	12 (50.0%)
Professional/graduate education	4 (16.7%)
**Annual household income**	
$0 - $20,000	1 (4.2%)
$21,000 - $60,000	3 (12.5%)
$61,000 - $90,000	6 (25.0%)
$91,000 - $120,000	2 (8.3%)
$121,000 - $150,000	4 (16.7%)
$151,000+	8 (33.3%)
Prefer not to say	0 (0.0%)

**Table 2.** Personal History Information

	n (%)
**Diagnosis of Li-Fraumeni syndrome**	
Yes	22 (91.7%)
Prenatal diagnosis	0 (0.0%)
0–5 years old at diagnosis	0 (0.0%)
6–12 years old at diagnosis	1 (4.5%)
13–17 years old at diagnosis	2 (9.1%)
18–25 years old at diagnosis	2 (9.1%)
26–40 years old at diagnosis	11 (50.0%)
41–65 years old at diagnosis	6 (27.3%)
66+ years old at diagnosis	0 (0.0%)
No	2 (8.3%)
**Diagnosis of cancer** ^a^	
Yes	14 (63.6%)
Age at diagnosis^b^	
Early Childhood (12 years and below)	2 (5.7%)
Adolescence (13–17 years old)	1 (2.9%)
Young-Middle Adulthood (18–45 years)	17 (48.6%)
Middle-Late Adulthood (46+)	15 (42.9%)
No	8 (36.4%)

a Question asked only to respondents who indicated a personal history of LFS

b All prior cancer diagnoses are listed, with some respondents reporting more than one cancer diagnosis

**Table 3.** Children’s Cancer History Information

	n (%)
**Number of biological children**	
0 children	8 (33.3%)
1 child	3 (12.5%)
2 children	8 (33.3%)
3 children	4 (16.7%)
4 children	0 (0.0%)
5 children	1 (4.2%)
6+ children	0 (0.0%)
Median number of children (IQR)	2 (0, 2)
**Proportion of children who have undergone genetic testing for LFS**	
All children	14 (87.5%)
Some children	2 (12.5%)
No children	0 (0.0%)
**Number of children with LFS diagnosis**	
0 children	4 (25.0%)
1 child	6 (37.5%)
2 children	4 (25.0%)
3 children	2 (12.5%)
4 children	0 (0.0%)
5 children	0 (0.0%)
6+ children	0 (0.0%)
Median number of children (IQR)	1 (0.5, 2)
**Mode of LFS inheritance**	
From an affected parent	12 (100.0%)
From an unaffected parent with germline mosaicism	0 (0.0%)
From an unaffected parent without germline mosaicism	0 (0.0%)
**Child age at diagnosis with LFS**	
Birth-5 years old at diagnosis	10 (50.0%)
6–12 years old at diagnosis	4 (20.0%)
13–17 years old at diagnosis	3 (15.0%)
18–25 years old at diagnosis	2 (10.0%)
26–40 years old at diagnosis	1 (5.0%)
41–65 years old at diagnosis	0 (0.0%)
66+ years old at diagnosis	0 (0.0%)
**Children with LFS without cancer**	9 (75.0%)
**Children with LFS and cancer**	3 (25.0%)
**Child age at first cancer diagnosis**	
Childhood (12 years and below)	3 (75%)
Adolescence (13–17 years old)	0 (0.0%)
18–45 years	1 (25%)
46+ years	0 (0.0%)
**Child cancer types**	
Choroid plexus carcinoma	1 (16.7%)
Non-differentiated sarcoma	1 (16.7%)
Rhabdomyosarcoma	2 (33.3%)
Retroperitoneal pleomorphic sarcoma	1 (16.7%)
Acute lymphoblastic leukemia	1 (16.7%)
**Timing of LFS diagnosis**	
LFS diagnosed before cancer	0 (0.0%)
Cancer diagnosed before LFS	4 (100.0%)

LFS: Li-Fraumeni syndrome

**Table 4.** Perspectives on Newborn Screening for LFS

	n (%)
In support of newborn screening for LFS	
Yes	24 (100.0%)
No	0 (0.0%)
Unsure	0 (0.0%)
**Beliefs regarding “having an answer” of child’s LFS status** (check all that apply)	
Screening would reduce uncertainty	16 (66.7%)
Screening would provide peace of mind	13 (54.2%)
Screening would provide a sense of control	16 (66.7%)
Knowledge is power	20 (83.3%)
None of the above	0 (0.0%)
**Feelings regarding the “emotional impact” of learning from newborn screening that child has LFS** (check all that apply)	
Relieved	1 (4.2%)
Happy	0 (0.0%)
Confused	1 (4.2%)
Guilty	10 (41.7%)
Anxious	15 (62.5%)
Depressed	7 (29.2%)
Shocked	4 (16.7%)
Angry	5 (20.8%)
Devastated	12 (50.0%)
None of the above	2 (8.3%)
**Feelings regarding the “emotional impact” of learning from newborn screening that child does not have LFS** (check all that apply)	
Relieved	24 (100.0%)
Happy	15 (62.5%)
Confused	0 (0.0%)
Guilty	1 (4.2%)
Anxious	0 (0.0%)
Depressed	1 (4.2%)
Shocked	1 (4.2%)
Angry	0 (0.0%)
Devastated	0 (0.0%)
None of the above	0 (0.0%)
**Beliefs regarding “knowledge as an opportunity”** (check all that apply)	
A positive result allows me to better prepare for my child’s future	18 (75.0%)
A positive result provides the opportunity for the most appropriate medical surveillance plan	22 (91.7%)
A positive result provides an opportunity for cancer prevention efforts	22 (91.7%)
Screening is in the best interest of my child	21 (87.5%)
Screening can provide information for future family planning	19 (79.2%)
Screening can contribute to research efforts	21 (87.5%)
None of the above	0 (0.0%)
**Beliefs regarding “knowledge as a barrier”** (check all that apply)	
Screening would impact the expectations I have for my child	5 (20.8%)
I would have difficulty communicating a positive result to family and friends	2 (8.3%)
A positive result would cause anxiety for my child	14 (58.3%)
Choosing to undergo screening would interfere with my child’s right to learn of their LFS status on their own	0 (0.0%)
None of the above	8 (33.3%)
**Concerns about newborn screening for LFS** (check all that apply)	
Insurance, privacy, and discrimination as it relates to screening for LFS	13 (54.2%)
Uncertain screening results	5 (20.8%)
The frequency of cancer surveillance procedures if my child screens positive	12 (50.0%)
The safety of cancer surveillance procedures if my child screens positive	6 (25.0%)
The financial cost of cancer surveillance procedures if my child screens positive	15 (62.5%)
The emotional burden of cancer surveillance procedures if my child screens positive	15 (62.5%)
None of the above	2 (8.3%)
**Parental consent should be required to screen for LFS on newborn screening**	
Yes	20 (83.3%)
No	0 (0.0%)
Unsure	4 (16.7%)

LFS: Li-Fraumeni syndrome
